# A simple and cheap aerosol penetrometer for filter testing using an electronic cigarette.

**DOI:** 10.12688/openreseurope.13087.1

**Published:** 2021-03-24

**Authors:** Sebastian Lifka, Ivan Ponomarev, Agnes Weth, David Baumgartner, Bernd Lamprecht, Werner Baumgartner

**Affiliations:** 1 Institute of Biomedical Mechatronics, Johannes Kepler University of Linz, Linz, 4040, Austria; 2 ELMARCO s.r.o., Liberec, 46010, Czech Republic; 3 Department of Pneumology, Kepler University Hospital Linz, Linz, 4020, Austria

**Keywords:** Filter, face masks, penetrometer, electronic cigarette, COVID-19, light scattering detector

## Abstract

**Background:** During the coronavirus disease 2019 (COVID-19) pandemic face masks grew in importance as their use by the general population was recommended by health officials in order to minimize the risk of infection and prevent further spread of the virus. To ensure health protection of medical personal and other system relevant staff, it is of considerable interest to quickly test if a certain lot of filtering facepiece masks meets the requirements or if the permeability changes under different conditions. As certified penetrometers are rather expensive and were difficult to obtain during the COVID-19 pandemic, we describe two quite simple and cheap methods to quickly test the filter permeability based on an electronic cigarette.

**Methods:** The first method uses a precision scale, the second method uses a light scattering detector to measure the filter penetration. To make sure these two methods yield reliable results, both were tested with freshly cut filter samples covering the range of approx. 2 % to 60 % permeability and compared to the results of a certified penetrometer.

**Results:** The comparison of the two methods with the certified penetrometer showed a good correlation and therefore allow a quick and rather reliable estimation of the permeability.

**Conclusions:** Several examples about the use of faulty masks and the resulting health risks show that simple, fast, cheap and broadly available methods for filter characterization might be useful in these days.

## Plain language summary

During the coronavirus disease 2019 (COVID-19) pandemic face masks grew in importance as their use by the general population was recommended by health officials in order to minimize the risk of infection and prevent further spread of the virus. High end face masks are important for health care professionals as well as professionals in other fields. To ensure health protection of medical personal and other system relevant staff, it is of considerable interest to quickly test if a certain lot of filtering facepiece masks meets the requirements or if the permeability changes under different conditions. The permeability for aerosols is usually quantified with a so-called penetrometer. In this device aerosol droplets of oily liquids are generated and the percentage of droplets which pass through the mask is measured. As certified penetrometers are rather expensive and were difficult to obtain during the COVID-19 pandemic, we describe two quite simple and cheap methods to quickly test the filter permeability based on an electronic cigarette. The first method uses a precision scale, the second method uses a light scattering detector built using only simple and cheap electronic components to measure the filter penetration. To make sure these two methods yield reliable results, both were tested with freshly cut filter samples covering a wide range of permeabilities and compared to the results of a certified high-end penetrometer. The comparison of the two methods with the certified penetrometer showed very similar results and therefore allow a quick and rather reliable estimation of the permeability. The methods were used to test different effects onto the quality of face masks. Several examples about the use of faulty masks and the resulting health risks show that simple, fast, cheap and broadly available methods for filter characterization might be useful in these days.

## Introduction

A particulate air filter is a device composed of fibrous, or porous materials which removes solid particulates such as dust, pollen, mold, and bacteria from the air. Air filters are of enormous importance in biomedical applications where the removal of pathogens from air is vital. Particular attention is paid during the coronavirus disease 2019 (COVID-19) pandemic to filtering half masks ( Bourouib, 2020; Li *et al.,* 2020; van Doremalen *et al.,* 2020; Zhang *et al.,* 2020).

A half mask encloses nose and mouth. The eye area is left out here so that it cannot be used without protective goggles in an environment with harmful substances that can cause irritation or damage to the eyes. Fields of application are, for example, dust protection masks for grinding work in the trade or on construction sites, for silo inspections, in mining or against aerosols, e.g. for paint application (exclusively water-based paints) with spray gun or airbrush. Furthermore, certain particle-filtering half masks are said to provide at least partial protection against various pathogens, such as severe acute respiratory syndrome coronavirus 2 (SARS-CoV-2) ( Li *et al.,* 2020; Zhang *et al.,* 2020). Zhang *et al.* (2020) found that non-medical mask-wearing by 75 % of the population reduced infections, hospitalizations, and deaths by about 40 %. Sheltering individuals aged 50 to 64 years of age when combined with mask-wearing decreasing attack rate, hospitalizations, and deaths by over 82%.

To contain the spread of SARS-CoV-2, face masks were used as a health protection measure during the COVID-19 pandemic ( Li *et al.,* 2020). To minimize the risk of transmission of the virus, the use of face masks by the general population has been recommended by health officials. In order to provide special protection to health care workers and other professionals who are inevitably exposed to dust and aerosols, medical grade face masks, such as N95 respirators, should be reserved for the aforementioned clientele ( NIOSH, 2003). Shortages of face masks led to some uncertified and substandard masks with significantly reduced performance being sold on the market ( Wang *et al.,* 2020).

Throughout the COVID-19-pandemic various types of face masks have been recommended including ( Bartoszko *et al.,* 2020; Douglas *et al.,* 2020; Wang *et al.,* 2020; Zhao *et al.,* 2020):

- cloth face masks
- loose-fitting medical or surgical masks
- face-sealing filtering facepiece masks, including uncertified dust masks as well as certified respirator masks (with respirator certifications such as N95 respirators, N99 respirators, and filtering face piece (FFP) respirators)
- other respirators, including elastomeric respirators, some of which may also be considered filtering facepieces.

Particle filtering face pieces (fine dust mask, dust mask or respiratory protection filter) protect against the inhalation of particles and aqueous or oily aerosols ( NIOSH, 2003). They do not provide protection against gases and vapors, even if they are equipped with an activated carbon insert. This inlay serves to protect against unpleasant but harmless organic odors (e.g. for handling slaughterhouse waste, in animal breeding or waste disposal). Filtering half-masks usually consist entirely of non-woven fabric with elastic bands and a moldable nose clip to optimize adaptation to the face. Correct usage is vital here, when used properly, the masks with test certification provide reliable protection against respirable dust and liquid mist within their respective application area. ( Degesys *et al.,* 2020; Regli *et al.,* 2021; Rollings, 2020). In addition to the supporting filter material, they have layers of an electrostatic material (electret, see also electret filter). Here, small dust particles and liquid drops are bound by electrostatic forces ( Romay *et al.,* 1998). However, the electrostatic effect, and therefore also the filter efficiency, decreases after some time due to dust accumulation, and the deposits also lead to a noticeable increase in breathing resistance ( Ji *et al.,* 2003). A classification of particle filtering face pieces is made according to the European standard ÖNORM EN, EN 149:2009 07 01, 2009, Ch. 7.9.1 and 7.9.2 in three classes, namely FFP1, FFP2 (KN95) and FFP3 (KN99). The assessment is based on the total inward leakage of a mask, which consists of leakage points on the face, the leakage at the exhalation valve (if present) and the penetration of the filter material.

**FFP1:** Maximum 25 % total inward leakage (mean values not greater than 22 %); maximum penetration of filter material of 20 % (corresponds to a filter percentage of 80 %); for non-toxic and non-fibrogenic dusts; maximum concentration up to 4 times the maximum workplace concentration.

( ÖNORM EN, EN 149:2009 07 01, 2009, Ch. 7.9.1 and 7.9.2)

**FFP2 (KN95):** Maximum 11 % total inward leakage (mean values not greater than 8 %); maximum penetration of filter material of 6 % (corresponds to a filter percentage of 94 %); for dusts, mists and smokes harmful to health; filters for solid and liquid particles; against harmful substances whose concentration is up to 10 times the maximum workplace concentration.

( ÖNORM EN, EN 149:2009 07 01, 2009, Ch. 7.9.1 and 7.9.2)

**FFP3 (KN99):** Maximum 5 % total inward leakage (mean values not greater than 2 %); maximum penetration of filter material of 1% (corresponds to a filter percentage of 99%); protection against toxic substances as well as against droplet aerosols, carcinogenic or radioactive substances, enzymes, microorganisms (viruses, bacteria, fungi and their spores); against harmful substances whose concentration ranges up to 30 times the maximum workplace concentration.

( ÖNORM EN, EN 149:2009 07 01, 2009, Ch. 7.9.1 and 7.9.2)

Medical oral and nasal protection (surgical face mask, medical face mask, clinical mask or hygiene mask) is a medical device with the purpose of reducing the transmission of pathogens through secretion droplets (droplet infection). This is a medical face half-mask, which is fixed to the back of the head or behind the ears with bandage or elastic bands. Professional oronasal masks are typically made of nonwoven fabric. They are usually made in three layers as "SMS" (spunbond-meltblown-spunbond)-laminates. This means that the two outer layers are spunbond materials and the layer in between is a nonwoven fabric produced by the meltblown process. The meltblown nonwoven layer in between consists of extremely small randomly arranged fibers that give the mask the required separation efficiency. An alternative to the meltblown nonwoven are electrospun nanofibers. Electrostatic effects of the middle nonwoven (meltblown or electrospun) are supposed to be particularly important to filter out small particles or aerosol droplets.

Besides these personal protection masks also High-efficiency particulate air (HEPA)-filters and other filters in air-conditions and air supply for keeping air clean in buildings or within chambers are of interest. To fulfill certain levels of efficiency, HEPA-filters must remove at least 99.95 % ( ÖNORM EN, EN 1822-1:2019, 2019) or 99.97 % ( DOE-STD-3020-2005, 2005) of particles having a diameter of 0.3 µm from the air passing through the filter. For particles with lower or higher diameter, the filter efficiency increases. Irving Langmuir identified particles with a diameter of 0.3 µm to be the most penetrating size particles, also called the “most penetrating particle size” (MPPS).

Thus, today the efficiency of filters is usually tested using a test aerosol in an aerosol-penetrometer. Typically, Bis(2-ethylhexyl) phthalate (dioctyl phthalate, DOP) or Di-2-ethylhexyl-sebacat (DEHS) are evaporated in a way to achieve droplets of the MPPS and the percentage of these droplets penetrating the filters is measured ( Carlon *et al.,* 1991). Certified penetrometers for filter testing are rather expensive and were hard to obtain during the COVID-19 pandemic. However, it is quite interesting to quickly test, whether a certain lot of filters is ok. Furthermore, it is of interest to determine if the permeability of filters changes under different conditions, for example if used over a certain period of time or under different conditions.

Here we describe a simple aerosol-penetrometer setup based on an electronic cigarette. These devices produce a highly concentrated inhalation aerosol by an evaporation/condensation process. They electrically heat a solution consisting primarily of propylene glycol, glycerol, and additives (nicotine, water and flavors). The hot vapor is then rapidly cooled as it is drawn through the device by the user, causing it to nucleate and condense into an aerosol. If done in a discontinuous way by applying “Puffs” of approximately 50 ml, similar to typical smoking behavior, these electronic cigarettes produce an aerosol with droplet sizes around 300 nm ( Ingebrethsen *et al.,* 2012; Kane & Li, 2020; Sosnowski & Odziomek, 2018), which is a size that can penetrate into the lung most effectively. We show two methods of quantification of this hot vapor that can be applied simple and cheaply in order to characterize filter behavior. 

## Methods

### Penetration measurement using a precision scale

**Figure 1.**
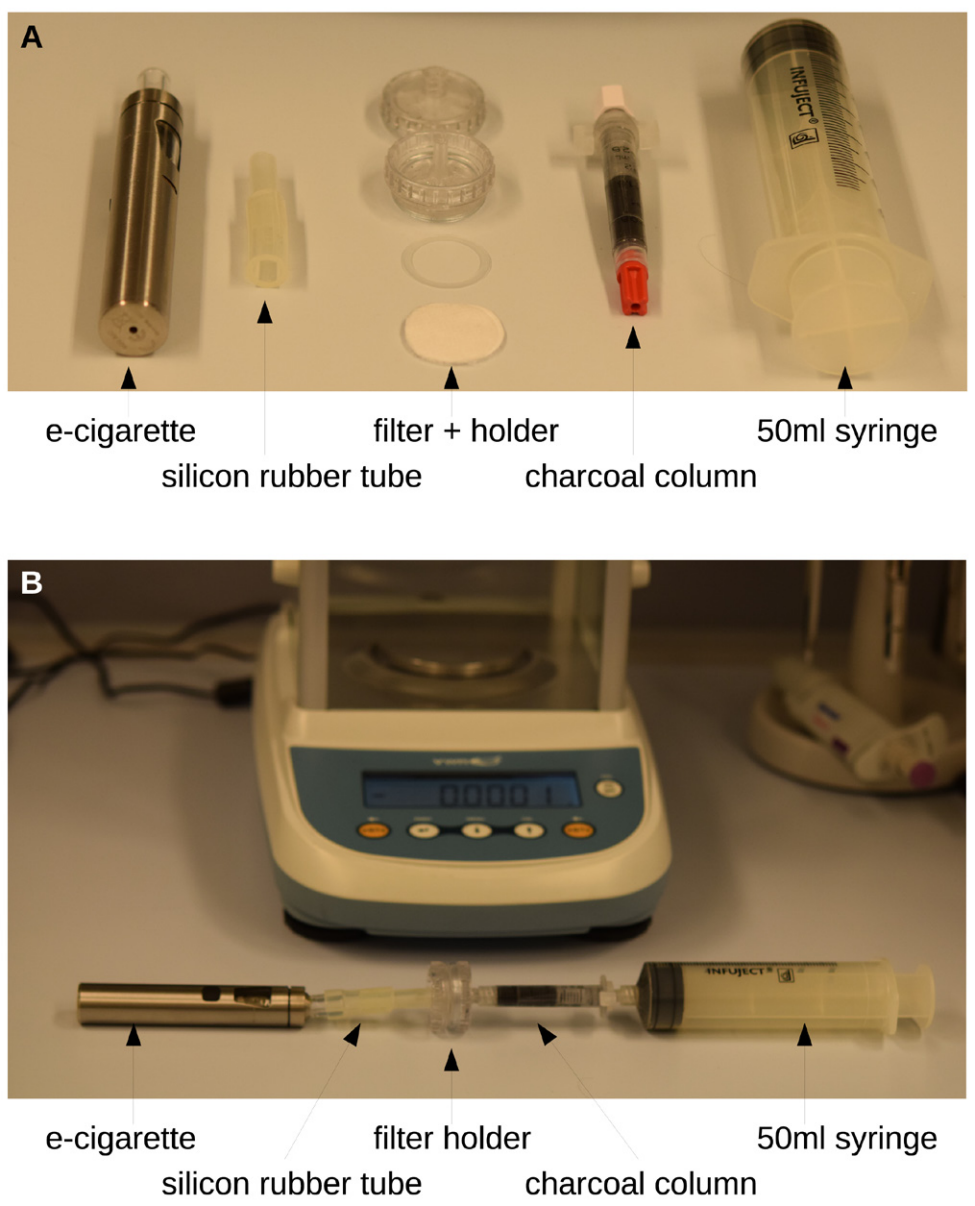
Setup for the scale-method. The single components ( **A**) are connected as shown in ( **B**). The evaporation was started by pressing the steamer button of the electronic cigarette and 50 ml were sucked up by the syringe within 2–5 s. The increase in weight of the charcoal-column was immediately determined by the precision scale.

An electronic cigarette (eGo AIO, Joytech, ShenZeng, China) equipped with a 0.6 Ω coil was filled with basic liquid (ZAZO Basis 10ml/0mg nicotine, catalogue number: Z00000001, ZAZO Intrade Concepts GmbH, Euskirchen, Germany) containing 1,2-propylene glycol (PG) and glycerol (Gly). By fitting silicone rubber hoses this was attached to a 2.5 cm polycarbonate syringe filter holder (Art. Nr. 16517, Sartorius, Göttingen, Germany). Discs with a diameter of approx. 24 mm of the filters under investigation were cut out of the face masks and placed into the filter holder. On the side with the Luer-lock of the filter holder a 2.5 ml column (Art. Nr. S1012, MoBiTec, Göttingen, Germany) was placed containing 0.5-0.8 g of fine charcoal (Art. Nr. C2764, Sigma, Vienna, Austria) between two frits. Finally, a 50 ml syringe (Infuject, Dispomed, Gelnhausen, Germany) was used to pump the aerosol from the electronic cigarette through the filter and into the charcoal. The setup is depicted in Figure 1.

The procedure of permeability determination was as follows. Initially the charcoal-column was weighed and the initial weight was tared to 0 on a precision scale (Kern ALS-A, Kern, Balingen, Germany). Then the setup was assembled as shown in Figure 1B. The evaporation was started by pressing the steamer button of the electronic cigarette and 50 ml were sucked up by the syringe within 2–5 s. Then the column was immediately removed from the setup and weighted again. The increase in weight of the charcoal-column was immediately determined by the precision scale. This was done initially with an empty filter holder to determine the amount of aerosol that is produced and can pass. Typically, this control value was 12mg. Then the filter was inserted into the holder and the measurement was repeated. The ratio of the measurement with the filter and the control value directly yields the permeability. For control reasons the measurement with the empty filter holder was repeated at the end of the measurement series. If the initial control value and the final control value differ significantly, the series has to be discarded. 

### Penetration measurement using a light scattering detector

**Figure 2.**
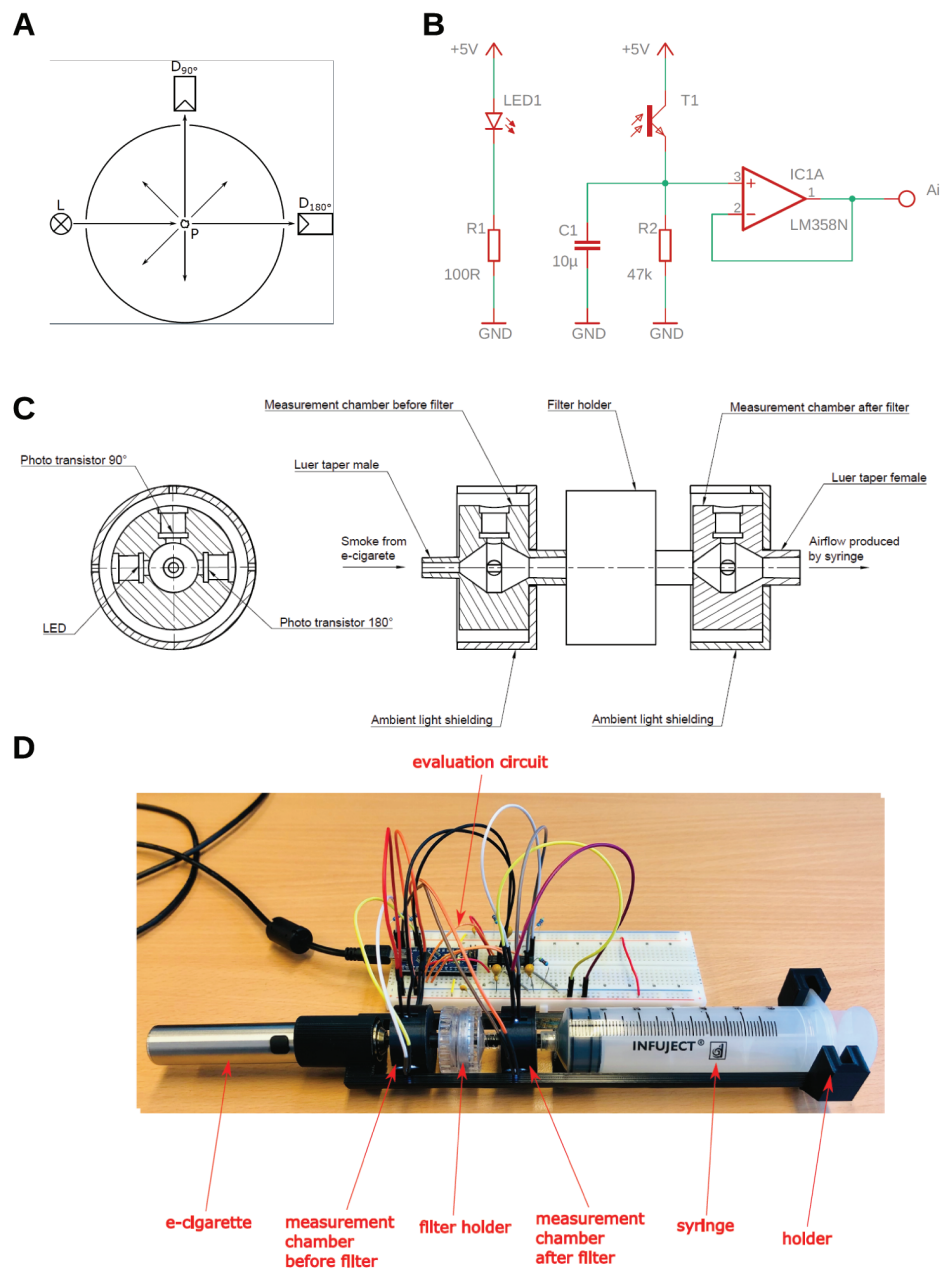
Setup of scatter method. The amount of aerosol-droplets is measured by light scattering. The scheme is shown in ( **A**). Phototransistors are placed at 180° and 90° to an LED respectively. The electronic control and readout are depicted in ( **B**). Care has to be taken to avoid leakage and stray light from outside. This an adequate housing was built shown in ( **C**). The whole arrangement is depicted in ( **D**). The outputs (Ai) of each individual amplifier circuit were read out using an Arduino micro-controller.

The setup is shown in Figure 2. Generally, the setup is identical to that one for the scale method, but the charcoal-column is removed and a light scatter detector is placed between the electronic cigarette and the filter holder and between the filter holder and the syringe respectively.

The amount of aerosol-droplets is measured by light scattering. Phototransistors (BPW96C, Vishay Semiconductors, Malvern, Pennsylvania, USA) are placed at 180° and 90° to a white LED with spectral maximum at 450 nm wavelength (C503D-WAN, Cree, Durham, North Carolina USA) respectively. If no droplets are present the light from the LED only reaches the detector at 180° (direct light) and no light reaches the detector at 90°. If droplets are present, the light from the LED is scattered at these droplets and a part of the light also reaches the detector at 90° (scattered light). The ratio between the scattered light and the direct light is a measure for the turbidity in the measurement chamber ( Kitchener *et al.,* 2017; Wang *et al.,* 2018). As one can see, the higher the turbidity in the measurement chamber, the more droplets are present. To measure the filter penetration, a measurement chamber with stray light detector was put before and after the filter holder.

The electronic control and readout are depicted in Figure 2B. As voltage source the 5 V supply voltage of the used Arduino micro-controller board (Arduino Nano) was used. A 100 Ω ±1 % series resistor was used for the LED which leads to a forward current of 18 mA. The LED was connected to the digital outputs D9 and D10 of the Arduino and powered with a pulse width modulation (PWM) of 100 %. Lower percentages of the PWM are not recommended, because the phototransistor is fast enough to measure the changes in luminous intensity. For amplification and conversion of the collector light current from the phototransistor into a voltage a 47 kΩ ±1 % series resistor was used. This potential is then applied to the input of an operational amplifier (LM358N, STMicroelectronic, Genf, Switzerland) which is connected as a voltage follower. The outputs (Ai) of each individual amplifier circuit were read out using the analog inputs A0 to A3 (built in 10 Bit analog digital converter) of the Arduino, which transferred the 4 measurements via the USB-Port to a computer. The Arduino code was written in the Arduino IDE 1.8.13.

Care has to be taken to avoid leakage and stray light from outside. Thus, an adequate housing was built by 3D-printing (Ender 3 Pro, Creality, Shenzhen, China) as shown in Figure 2C. The electronic cigarette, the measurement chambers and the filter holder are connected over a Luer system. For easier handling a holder for the whole arrangement was designed. The holder and the measurement chambers were also manufactured by means of 3D-printing. To ensure good air sealing in the area where the LEDs and the phototransistor are inserted into the measurement chamber, two seal rings per LED/phototransistor were used. To fit the electronic cigarette onto the measurement chamber, an additional adapter was designed and manufactured by means of 3D-printing. Again, to ensure good air sealing two sealing rings were used with this adapter. All CAD files used are available as extended data ( Lifka, 2021).

The code for measurement evaluation was written in Python 3.8 with Spyder IDE 4.1.5. The measured values were recorded for 30 s and the ratio of the scattered light (90°-phototransistor) and the direct light (180°-phototransistor) was determined. Initially these ratios were recorded for 15 s without hot vapor from the electronic cigarette yielding the base line. Then the evaporation was started by pressing the steamer button of the electronic cigarette and 50 ml were sucked up by the syringe within 2–5 s. The light-ratio was recorded for 30 s and finally the turbidity before and after the filter were integrated using Newtons integration method. The ratio of the integral values yields the permeability of the filter used. The whole arrangement is shown in Figure 2D. Both the Python code and the Arduino code used are available as extended data ( Lifka, 2021).

### Comparisons

Different commercially available filters of different default permeability were taken. These were a spunbond + nanofibers (short Spunbond) produced by Elmarco (Liberce, Czech Republic), a simple cotton mask (short Cotton, Elmarco), the combination of the cotton and the spunbond/nanofibre material (short Spunbond + Cotton), a simple face mask CUBO (NANOzLiberece, Czech Republic), a meltblown surgical facemask and a KN95 (short KN95, CareAble Biotechnology Co., Ltd, China), and a FFP2-Prototype (short FFP2, Lenzing AG, Lenzing, Austria). 

For comparison with our methods a certified penetrometer was used according to the manufacturer’s recommendations. This was an ATI Penetrometer TDA-100P with a flow of 30 l/min through 100 cm ^2^ and droplet size 0.3 µm using Bis(2-ethylhexyl) phthalate (DOP). 

To analyze the time behavior of the filter penetration, additional measurements of the filter samples after being used or being discharged were performed and compared to the results of the measurements of the fresh samples.

For discharging of the filter material, the filters were subjected for 10 min to isopropanol-vapor. This was done by putting the filter into a covered glass-box which also contained a petri dish with 5 ml of isopropanol. Alternatively, KN95-masks were adjusted correctly to the face and used for 4 h continuously by a 49-year-old 95 kg male experimenter with a beard.

### Analysis

For the comparison of the scale and the light scattering method with the certified penetrometer, a measurement series with regard to filter penetration of three respectively six fresh cut samples from every filter to test was performed. For the statistical analysis, the mean value and the standard deviation of every measurement series were calculated.

For the analysis of the relative change of the permeability of different filters after being discharged, again a measurement series of three samples of every filter was performed and the mean values and the standard deviation of every measurement series was calculated.

All statistical analysis were performed with Python 3.8.5 (numpy 1.19.5). The visualization of the results were also performed with Python 3.8.5 (matplotlib 3.3.3).

## Results

**Figure 3.**
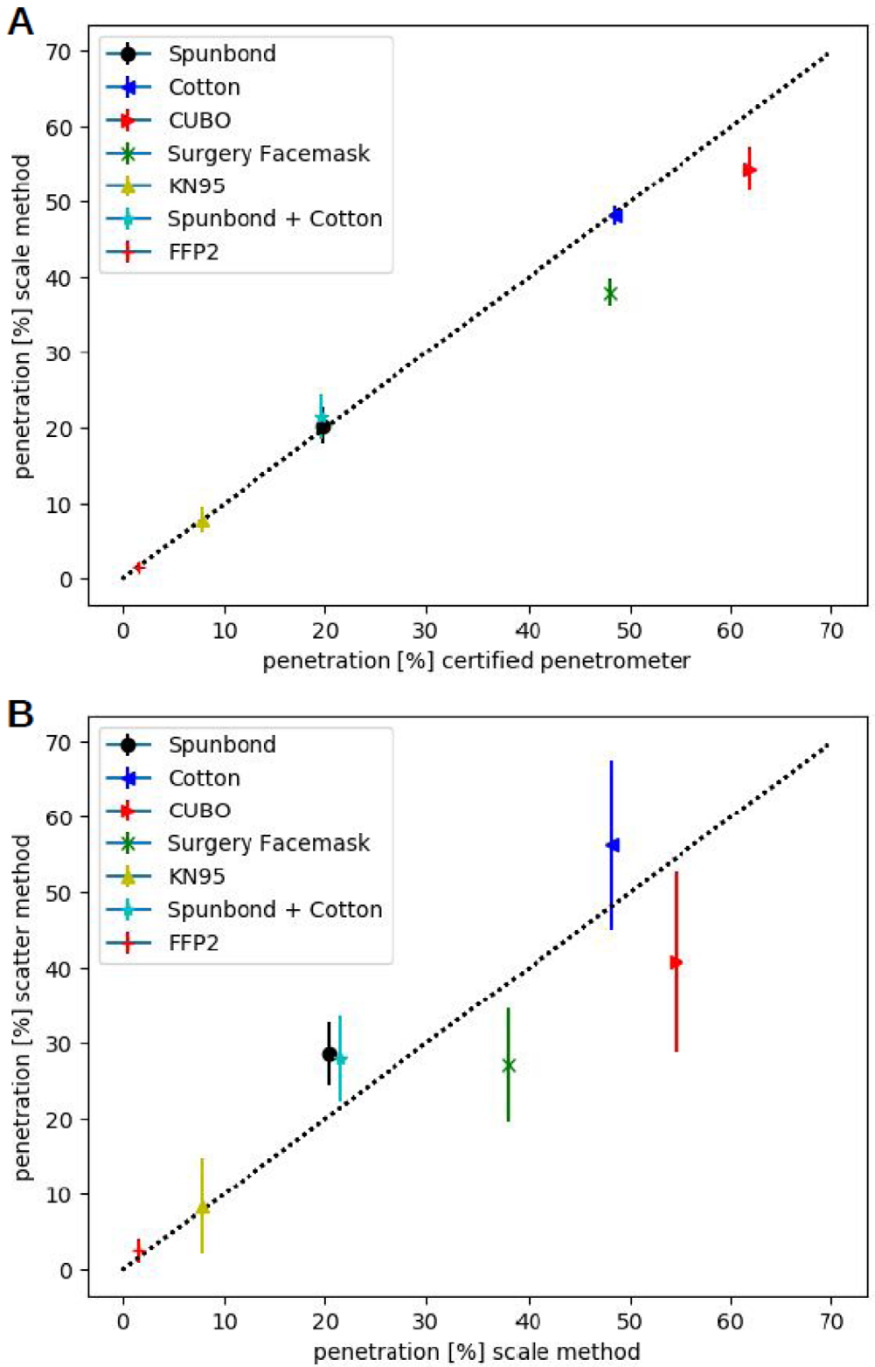
Comparison of the methods using different face filters. In ( **A**) the measured permeabilities using the scale method for different filters is plotted against the corresponding permeabilities determined using the certified ATI Penetrometer. The line with slope one through the origin is indicated as dotted line. Each measurement was repeated three times and the results are shown as mean and the error-bar represents the standard deviation. In ( **B**) the results of the scatter method are shown. Here each experiment was repeated 6 times. Also, here the mean values are shown and the error-bars depict the standard deviation.

To see if our methods, i.e. the scale-method and the scatter method yield meaningful results, both methods were tested with freshly cut samples from commercially available filters covering the range of approx. 2 % to 60 % permeability. The results are shown in Figure 3.

Clearly there is a good correlation of both methods with the measurements using the certified ATI Penetrometer. The scatter method has slightly higher variance in between the individual measurements, especially in the cases of higher permeability but, nonetheless, allows for a quick and rather reliable estimation of the permeability.

We observed that how quickly the syringe is drawn up is irrelevant as long as one stays within a time window of 1–5 s for the procedure.

**Figure 4.**
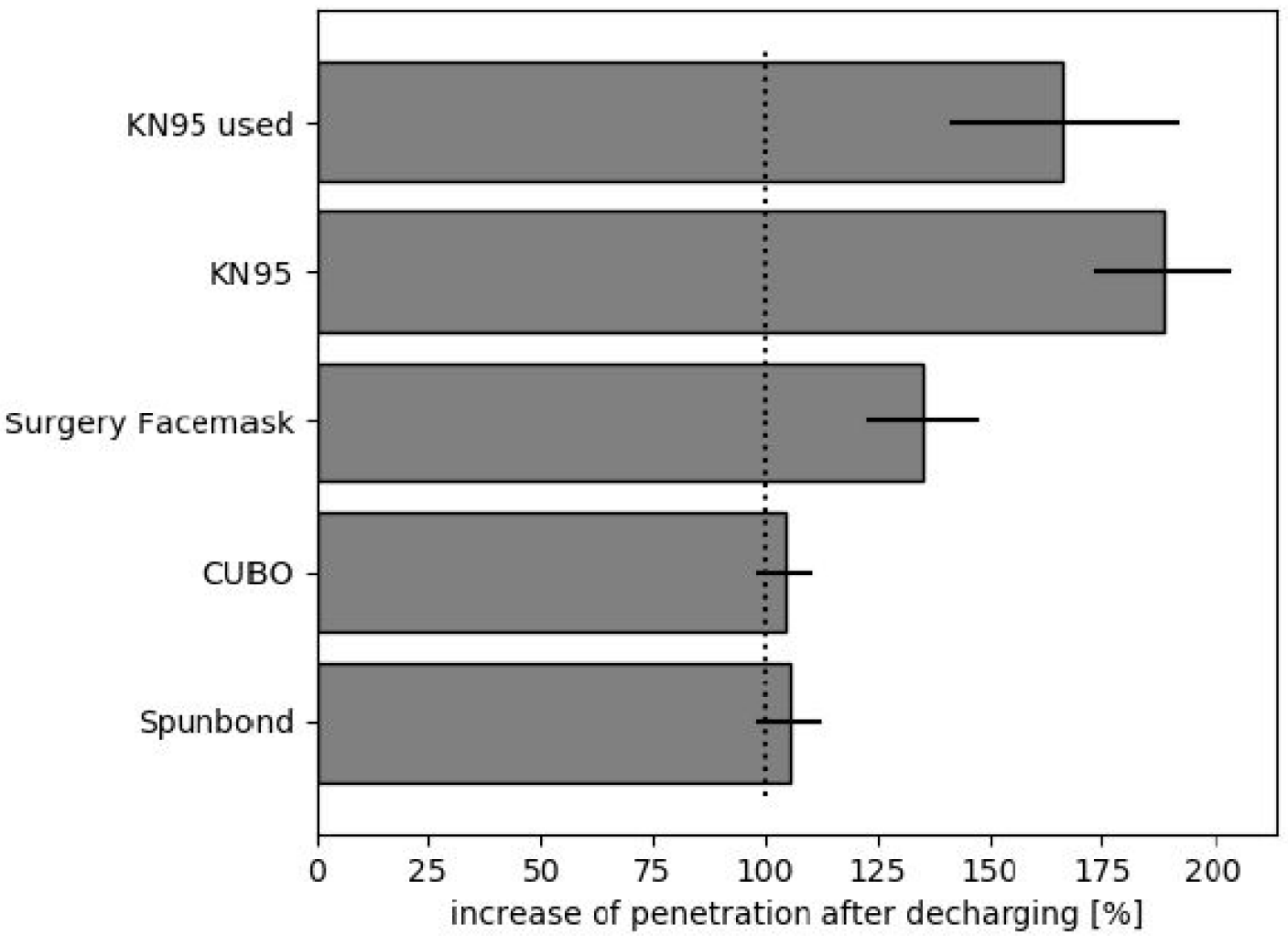
Relative change of the permeability of different filters after being discharged. The initial permeabilities of the different filters was set to 100 %. “KN95 used” shows the relative permeability of the KN95-filter after being worn for 4 h. All other filters were discharged by application of isopropanol vapor. For all conditions n=3.

To show that the method can be of use for the monitoring of filter behavior over time and under different conditions we measured the permeability of the filter after being discharged in comparison to a fresh filter. The results are depicted in Figure 4.

In this figure the initial permeabilities of the filters under investigation were set to 100 %. It is well known that some filter materials are treated to be an electret, i.e. to carry “permanent” charges. The electrostatic charge is the most efficient filtering mechanism therefore aerosol droplets are attracted by filtering fibers. The charges can be neutralized over time which should result in increased permeability. KN95-masks were worn for 4 h to simulate charge-neutralization by moisture in the breath (KN95 used). Furthermore KN95-material and other filter materials were discharged by applying isopropanol vapor. Clearly the filters that rely on meltblown nonwoven are rather vulnerable to discharging, while nanofibers pretty much keep their filter efficiency.

## Discussion

In the current manuscript we describe two methods for measuring the aerosol-penetration through filter material using an electronic cigarette. These electronic cigarettes produce droplets of about 300 nm diameter, which corresponds to the most penetrating particle size ( Agranovski, 2010; Sosnowski & Odziomek, 2018). Droplets of this diameter are the most difficult to remove and are thus used for aerosol penetrometers. It was shown that electronic cigarettes when driven by 50 ml puffs (and not in a continuous flow) produce exactly such droplets ( Kane & Li, 2020; Ingebrethsen *et al.,* 2012).

A simple electronic cigarette costs about €30. If a precision scale, which allows weighting with a resolution of 0.1 mg, is available in a lab, the cheap and precise scale method can be used. Here the penetrating aerosol is absorbed by a charcoal column and the weight difference is determined. The ratio of the absorbed aerosol with and without filter is taken as measure for the permeability. This method was found to be reliable, reproducible, simple to perform and does not require expensive equipment besides a precision scale.

To allow even broader use we developed a second approach which determines the ratio of aerosol particles before and after a filter by means of light scattering. Such a device requires only a few cheap electronic components and an Arduino micro controller. The total costs of the electronic components were in our case about €12. A holder can be simply manufactured, for example by means of 3D-printing using a standard extrusion printer. Especially in the case of low permeability, this method also yields good results and does not require access to an expensive precision scale.

We observed that how quickly the syringe is drawn up is irrelevant as long as one stays within a time window of 1–5 s for the procedure. This time window guarantees that the evaporation and condensation in the electronic cigarette takes place correctly and no significant evaporation of the aerosol droplets takes place before penetrating the filter under investigation.

The developed methods can help to monitor the quality of the filter material of face masks under different conditions and also allows the monitoring of the time behavior during use and the effect of re usage ( Fisher & Shaffer, 2014)

**Figure 5.**
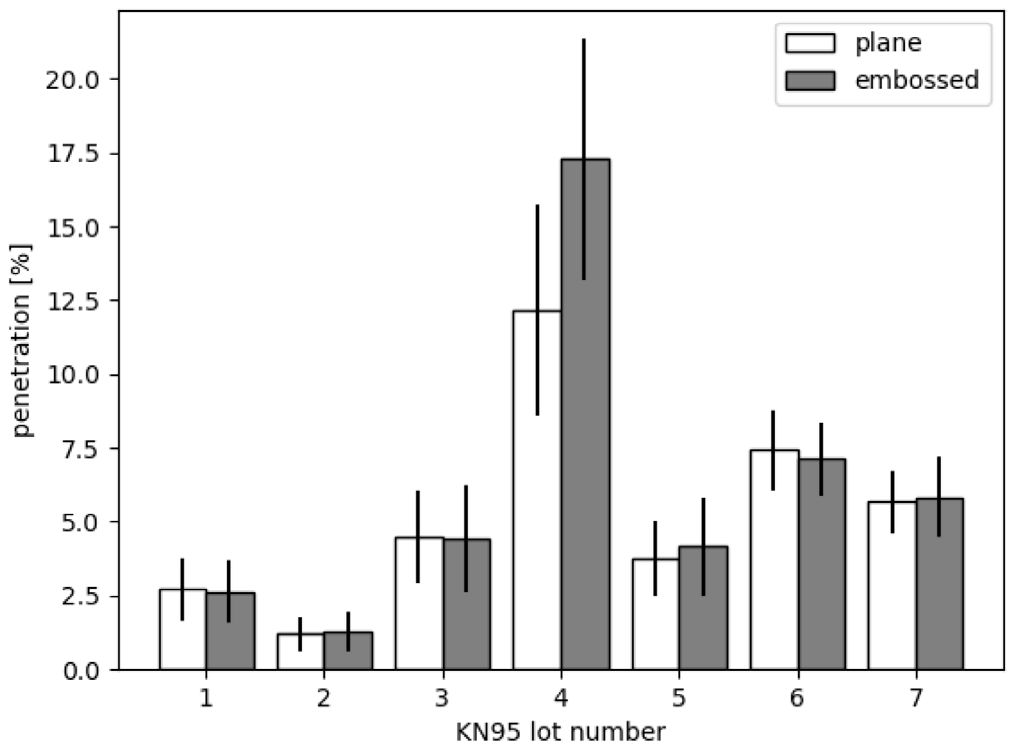
Permeabilities of different lots of KN95 face masks. Samples were taken from areas where the filter material is plaine (white) and from areas where lines for folding were embossed (gray). One can see that lot 4 was not ok and had a significant vulnerability within the embossed lines.

As there was an enormous demand for face masks during the COVID-19 pandemic, especially FFP2 (KN95) or even higher quality, some local traders got offered to buy face masks for which they had doubts about the quality despite the existence of all certificates. Therefore, we were asked by local traders to check samples where the quality was doubted using the methods presented. We could measure samples of the lots as depicted in Figure 5.

Interestingly most of the lots were within the tolerances. However, lot 4 was not ok. Even more interesting we could observe that the embossing of lines for folding the masks in order to fit tightly to the face can make a difference. While most of the masks show no increased permeability when taking a sample with such an embossed line, the masks from lot 4 had a significant vulnerability. Obviously, an insecure embossing method was used increasing the permeability up to intolerable levels.

This also shows that simple, fast, cheap and broadly available methods for the characterization of filters might be useful in these days. Applications for other air filters can be envisioned. One further improvement that could open the field to other applications would be the construction of an adapter capable of clamping the intact filter without the need to cut the filter into small samples. This would be easy to build.

## Data availability

### Underlying data

Zenodo: AerosolPenetrometer_LightScatteringDetector. http://doi.org/10.5281/zenodo.4541055 ( Lifka, 2021)

This project contains the following underlying data:

- Underlying_Data.xlsx (Excel file containing the underlying data of the results presented in figure 3 to 5)

### Extended data

Zenodo: AerosolPenetrometer_LightScatteringDetector. http://doi.org/10.5281/zenodo.4541055 ( Lifka, 2021)

This project contains the following extended data:

- Holder.stp (CAD file for the holder of the setup)
- Ambient_Light_Shielding.stp (CAD file of the ambient light shielding)
- Measurement_Chamber.stp (CAD file of the measurement chambers)
- Electronic_Cigarette_Adapter.stp (CAD file for the electronic cigarette adapter)
- Aerosol_Penetrometer_Light_Scattering_Detector_Arduino.ino (Arduino program for the light scattering detector)
- Aerosol_Penetrometer_Light_Scattering_Detector.py (Python class file for the light scattering detector)
- Aerosol_Penetrometer_Light_Scattering_Detector_Measurement_Script.py (Python measurement script for the light scattering detector)
- Measurement_Principal.pdf (Illustration of the measurement principle)
- Electronics.pdf (Electronic circuitry of the light scattering detector)
- Setup.pdf (Illustration of the measurement setup)
- Setup_Picture.pdf (Picture of the measurement setup)

Data are available under the terms of the Creative Commons Attribution 1.0 Generic license (CC-BY 1.0).
