# Supplementary material for: A simple and cheap aerosol penetrometer for filter testing using an electronic cigarette
Source: Open Res Eur. 2021 Oct 28;1:5. Originally published 2021 Mar 24. [Version 3] doi: 10.12688/openreseurope.13087.3 (PMC10445814; doi:10.12688/openreseurope.13087.3)
Supplement: Peer review history

# Peer review: Reviewer response for version 2

**DOI:** 10.21956/openreseurope.14923.r27211

**Published:** 2021-10-26

**Authors:** Clothilde Brochot

**Affiliations:** 1 Department of Building, Civil and Environmental Engineering, Concordia University, Montreal, Canada

The reviewer appreciated that the authors responded to all comments in your manuscript. This one has become clearer.

Is the study design appropriate and does the work have academic merit?

Partly

Is the work clearly and accurately presented and does it cite the current literature?

Partly

If applicable, is the statistical analysis and its interpretation appropriate?

Partly

Are all the source data underlying the results available to ensure full reproducibility?

Partly

Are the conclusions drawn adequately supported by the results?

Partly

Are sufficient details of methods and analysis provided to allow replication by others?

Partly

Reviewer Expertise:

Aerosol, filtration, filtering facepiece respirators, respiratory protective devices, filters for general ventilation.

I confirm that I have read this submission and believe that I have an appropriate level of expertise to confirm that it is of an acceptable scientific standard.

# Peer review: Reviewer response for version 2

**DOI:** 10.21956/openreseurope.14923.r27470

**Published:** 2021-09-20

**Authors:** Maria Dinescu

**Affiliations:** 1 National Institute for Lasers, Plasma and Radiation Physics, Măgurele, Romania

The paper presented by Lifka S. *et al.*, as it has been pointed out also by the previous reviewer, deals with the application of two methods, i.e. a weighting scale vs a light scattering photodetector to measure the filtration potential of different face masks against aerosol from electronic cigarettes.

 The revised version of the manuscript, where the authors have inserted all the comments suggested by the first reviewer, is clear, well organized, with conclusions supported by data. The work presented by S. Lifka *et al. *is very important not only for the medical community but also for the scientific community, i.e. for physicists, chemists, materials scientists, etc.

 The only comments, suggestions I have are related to little typos, for example, “Plain language summary” – “health protection of medical personnel (not personal)”. In addition, I would suggest the authors to insert more keywords (ex. face mask quality, particle filter, filter penetration measurement, etc.), in order for their paper to have a larger visibility.

Is the study design appropriate and does the work have academic merit?

Yes

Is the work clearly and accurately presented and does it cite the current literature?

Yes

If applicable, is the statistical analysis and its interpretation appropriate?

Yes

Are all the source data underlying the results available to ensure full reproducibility?

Yes

Are the conclusions drawn adequately supported by the results?

Yes

Are sufficient details of methods and analysis provided to allow replication by others?

Yes

Reviewer Expertise:

Laser processing, Optics, Functional coatings.

I confirm that I have read this submission and believe that I have an appropriate level of expertise to confirm that it is of an acceptable scientific standard.

# Peer review: Reviewer response for version 1

**DOI:** 10.21956/openreseurope.14157.r26858

**Published:** 2021-05-27

**Authors:** Clothilde Brochot

**Affiliations:** 1 Department of Building, Civil and Environmental Engineering, Concordia University, Montreal, Canada

This paper briefly presents two methodologies to measure the filtration performance of ‘mask’ samples (from face coverings to filtering facepiece respirators), against an aerosol generated by an electronic cigarette. The first method, by weighing, and based on a mass measurement, and the second, by photodetection, and also based on a mass measurement. These two methods are compared with a commercial measurement bench (ATI Penetrometer). The results presented seem to show a good consistency. The study presents interesting data; however, some limitations should be mentioned, and some concerns should be addressed to the authors.

 The reviewer would then have some revisions to propose (see below).

**Use of appropriate terms:**

 Although the work is presented in a structured manner, the use of the inappropriate terms makes the document difficult to read. Here are some quick definitions that may help authors replace terms in their paper.

 Penetrometer: name of the instrument for measuring the pressure drop and efficiency of a filter sample.

 Penetration: given as a function of the concentration downstream and upstream of a sample (filter or FFR).

 Permeability: The permeability of a medium is its ability to be crossed by a fluid under the effect of a pressure gradient (i.e. linked to the pressure drop and not i.e. efficiency). This quantity is related to Darcy's law.

 Respiratory Protective Device, Half mask respirator, Filtering Facepiece Respirator, Community masks, etc.

**More specific presentation of the standards cited in the paper:**

 The differences in the standards, especially between the European and the Chinese standards, do not allow to put in brackets the KN95 next to FFP2, nor the KN99 next to FFP3.

 Also, it would be good to quote the different parameters used in the standards. For example, the flow rate.

 Moreover, several parameters in the standards are well defined (and fixed), which is not the case in this study. Indeed, in this paper, no information is presented on the filtration flow rate, or velocity, yet this parameter has a considerable influence on the medium efficiency.

 In the same manner, the test aerosol used for the standards is well defined, centered at 0.3 micrometers (expressed in mass) with a defined maximum geometric standard deviation. The morphology of the particles used is also well known (spherical for oil and cubic for NaCl). The aerosol is brought to Boltzmann equilibrium, which is especially important during tests with electret media. A discussion of the different parameters used here should therefore be added in this paper.

**Parameters for the development of a measurement method:**

 To validate their measurement method, the authors performed a comparison with an established method. However, in addition to having well-determined parameters for the proposed method and with the ATI penetrometer (see comments on standards), the stability, reproducibility and accuracy of this method must be verified.

 In addition, some tests were performed on 3 samples and others on 6 samples. The authors should explain their choice in this difference. Also, normative tests usually take 10 samples for their tests, and scientific studies on filtering facepiece respirators use rather 5 samples to calculate their average performance.

Is the study design appropriate and does the work have academic merit?

Partly

Is the work clearly and accurately presented and does it cite the current literature?

Partly

If applicable, is the statistical analysis and its interpretation appropriate?

Partly

Are all the source data underlying the results available to ensure full reproducibility?

Partly

Are the conclusions drawn adequately supported by the results?

Partly

Are sufficient details of methods and analysis provided to allow replication by others?

Partly

Reviewer Expertise:

Aerosol, filtration, filtering facepiece respirators, respiratory protective devices, filters for general ventilation.

I confirm that I have read this submission and believe that I have an appropriate level of expertise to confirm that it is of an acceptable scientific standard, however I have significant reservations, as outlined above.

# Response

**Published:** 2021-07-02

**Authors:** Sebastian Lifka

**Affiliations:** Johannes Kepler University of Linz, Austria

We would like to thank the reviewer for her expertise and suggestions for improvements. In order to enhance our manuscript, we tried to implement the suggested improvements.

**Use of appropriate terms:**

- We rephrased some text passages in order to use appropriate terms, especially we considered the difference between permeability and filter penetration.

**More specific presentation of the standards cited in the paper:**

- We tried to present the standards used in this paper more precisely, especially we mentioned the difference between the European and the Chinese standard. Furthermore, we briefly discussed several parameters used in the European standard.

**Parameters for the development of a measurement method:**

- Due to the manual drawing up process of a syringe, the measurement parameters like flow rate or velocity can hardly be determined as well as prescribed in the presented standards. So of course, this affects the accuracy, reproducibility and stability of our presented methods. Therefore, we do not claim to reach the performance of a certified ATI-penetrometer. Regarding the difference in the sample number between the scale method (three samples) and the scatter method (six samples), the reason for this discrepancy is the higher variation of the measurement results of the scatter method, therefore we decided to use the double number of samples. To be consistent, we performed the measurement series for the new version of the light scattering detector again using six samples of each filter mask.
